# Molecular Evolution and Genomic Insights into Community-Acquired Methicillin-Resistant Staphylococcus aureus Sequence Type 88

**DOI:** 10.1128/spectrum.00342-22

**Published:** 2022-06-22

**Authors:** Wei Wang, Hui Li, Menghan Li, Yinping Dong, Yao Bai, Fengqin Li, Jing Xiao

**Affiliations:** a NHC Key Laboratory of Food Safety Risk Assessment, China National Center for Food Safety Risk Assessment, Beijing, China; b Division IV of Food Safety Standards, China National Center for Food Safety Risk Assessment, Beijing, China; University of West London

**Keywords:** MRSA, ST88, antimicrobial resistance, virulence factors, evolution, genome sequencing

## Abstract

Sequence type 88 (ST88) methicillin-resistant Staphylococcus aureus (MRSA) has been recognized as an important pathogen that causes infections in humans, especially when it has strong biofilm production and multidrug resistance (MDR). However, knowledge of the determinants of resistance or virulence and genomic characteristics of ST88 MRSA from China is still limited. In this study, we employed the antimicrobial resistance (AMR), biofilm formation, and genomic characteristics of ST88 MRSA collected from various foods in China and estimated the worldwide divergence of ST88 MRSA with publicly available ST88 genomes. All ST88 isolates studied were identified as having resistance genes, while 50% (41/82) harbored MDR genes. All isolates carried core virulence genes related to immune modulation, adherence, secreted enzymes, and hemolysin. In addition, all 20 Chinese ST88 isolates showed biofilm production capacity, three strongly so. Bayesian phylogenetic analysis showed that Chinese ST88 clones formed an independent MRSA lineage, with two subclades associated with acquisition of type IVc staphylococcal cassette chromosome *mec* (SCC*mec*) elements. In contrast, all African ST88 strains were subtyped as SCC*mec*IVa, where the African clades were mixed with a few European and American isolates, suggesting potential transmission from Africa to these regions. In summary, our results revealed the evolution of ST88 MRSA in humans, animals, and foods in Africa and Asia. The food-associated ST88 genomes in this study will remedy the lack of food-associated ST88 isolates, and the study in general will extend the discussion of the potential exchanges of ST88 between humans and foods or food animals.

**IMPORTANCE** ST88 MRSA has frequently been detected in humans, animals, and foods mainly in Africa and Asia. It can colonize and cause mild to severe infections in humans, especially children. Several studies from African countries have described its genotypic characteristics but, limited information is available on the evolution and characterization of ST88 MRSA in Asia, especially China. Meanwhile, the molecular history of its global spread remains largely unclear. In this study, we analyzed the genomic evolution of global ST88 MRSA strains in detail and identified key genetic changes associated with specific hosts or regions. Our results suggested geographical differentiation between ST88 MRSA’s evolution in Africa and its evolution in Asia, with a more recent clonal evolution in China. The introduction of ST88 MRSA in China was aligned with the acquisition of SCC*mec*IVc elements, specific virulent prophages, and AMR genes.

## INTRODUCTION

Staphylococcus aureus is a major foodborne pathogen that can produce a wide range of toxins including staphylococcal enterotoxins (SEs), which cause staphylococcal food poisoning (SFP) outbreaks (1). It is worth noting that frequent outbreaks of SFP have severely challenged public health, the food industry, and catering businesses (2, 3). In the United States, S. aureus causes more than 240,000 cases of foodborne illnesses each year (4). In Europe, in 2012, SFP was responsible for 346 foodborne outbreaks (FBOs), representing 6.4% of total outbreaks reported (5). In China, during 2003 and 2007, 94 FBOs were attributed to SFP that infected 2,223 individual patients and led to 1,186 hospitalizations (6). However, the actual incidence rate of SFP could be much higher because sporadic cases are easily overlooked and unreportable.

S. aureus has an extraordinary capacity for acquiring new antimicrobial resistance (AMR) genes (7, 8). The emergence and dissemination of multidrug-resistant (MDR) S. aureus, especially methicillin-resistant S. aureus (MRSA), has become a global concern and a leading cause of infections in both humans and animals (9). Since community-acquired MRSA (CA-MRSA) was first reported in the late 1990s, infections caused by MRSA are no longer confined to hospitals (hospital-acquired MRSA [HA-MRSA]) (10, 11). The above-mentioned factors can lead to difficulty in treating infections; prolonged hospitalization; increased health care costs; and the risk of AMR spreading to human communities, environment, or food media (12, 13).

Several geographically different lineages are associated with CA-MRSA infections, of which the clonal complexes (CCs) CC1, CC8, CC30, CC59, and CC80 are the most prevalent (14). However, the determinants of resistance or virulence that lead to the success of these MRSA lineages in the community are not fully understood. The sequence type 88 (ST88) MRSA lineage is mostly restricted to Africa and Asia but sporadically present elsewhere in the world (15, 16). Many African studies have identified it as a major circulating clone in hospital and community environments (17). Globally and in China, this clone has been identified from infections in human and veterinary hospitals (18–21). ST88 MRSA isolates usually contain the type IV or V staphylococcal cassette chromosome *mec* (SCC*mec*) elements, with or without Panton-Valentine leukocidin (PVL) (22). A recent study of clinical MRSA infections indicated that ST88 MRSA might be a strong biofilm producer, which poses a huge challenge for antimicrobial treatment (18).

ST88 MRSA has been recently detected in foods of animal origin, such as raw duck, chicken, fried diced meat, and dumplings containing meat (23–25). However, knowledge about determinants of resistance or virulence and genomic characteristics of food- and human-associated ST88 MRSA clones is still limited. The aim of this study was to unveil the genomic evolution of ST88 MRSA and to probe the genetic characteristics associated with its adaptation to different hosts and its AMR and virulence profiles.

## RESULTS

### General characteristics of ST88 strains.

**FIG 1:**
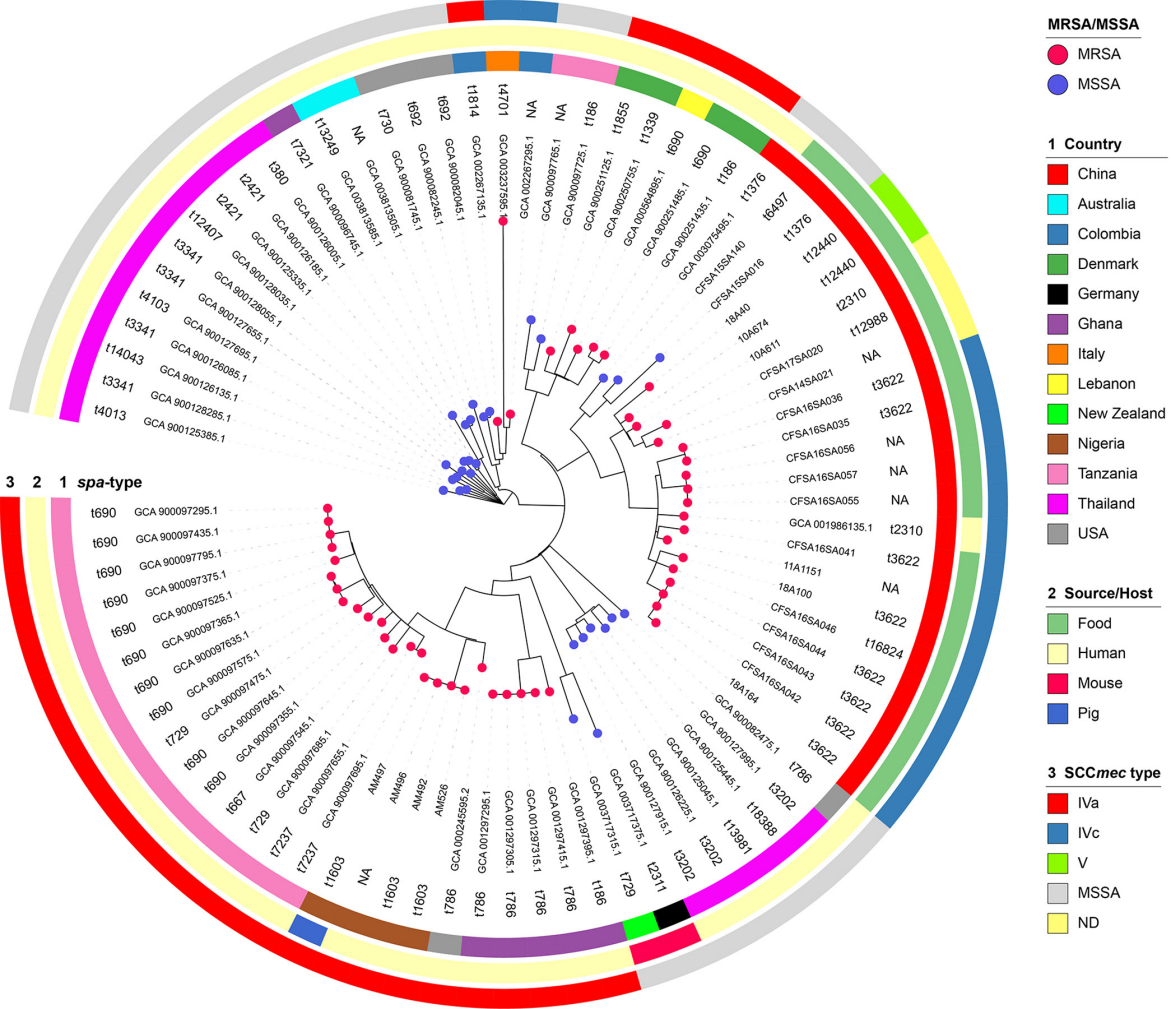
Maximum likelihood phylogenetic tree based on 2,300 core genes of the 82 ST88 S. aureus isolates from our study combined with publicly available isolates. Methicillin-resistant and -susceptible S. aureus isolates are indicated as red-pink (MRSA) or warm blue (MSSA) leaf nodes, respectively. The *spa* types, countries, sources/hosts, and SCC*mec* types are labeled or color coded in the following rings.

To explore the molecular evolution of ST88 strains, we analyzed publicly available genomes during the time of the study (June 2021; *n* = 62) combined with 20 genomes from our collection originating in animal-derived foods from eight provinces in China. These 82 ST88 strains were collected globally during 2008 to 2018 from human (*n* = 59), food (*n* = 20), and animal (*n* = 3) sources. These sources were mostly in China (*n* = 22), followed by Tanzania (*n* = 17), Thailand (*n* = 16), Ghana (*n* = 6), the United States (*n* = 5), and other countries (*n* ≤ 5 isolates per country) (see Fig. S1 and Data Set S1 in the supplemental material). All 82 isolates belonged to 33 *spa* types, with t690 the most dominant (12/82, 14.6%), followed by t786 (6/82, 7.3%). The predominant *spa* type in food-associated isolates was t3622 (8/20, 40%), all relevant samples of which were from China (Table 1, Fig. 1, and Data Set S1).

**TABLE 1:** Characteristics of human, food, and animal ST88 isolates

Characteristic	No. of isolates			
	Human (*n* = 59)	Food (*n* = 20)	Animal (*n* = 3)	Total (*n* = 82)
Region				
Asia (China, Thailand, and Lebanon)	19	20	0	39
Africa (Tanzania, Ghana, and Nigeria)	26	0	1	27
America (USA and Colombia)	7	0	0	7
Europe (Denmark, Germany, and Italy)	5	0	1	6
Oceania (Australia and New Zealand)	2	0	1	3
Major prophages				
Staphy_StauST398_3	49	20	3	72
Staphy_PT1028	35	20	1	56
Staphy_P282	31	20	2	53
Major prophages carrying virulence factors				
Staphy_P282 carrying IEC-1 *(scn*-*chp*-*sak*)	31	20	2	53
Staphy_phi2958PVL carrying PVL (*lukF*-*lukS*)	19	1	0	20
Major *spa* type				
t690	12	0	0	12
t3622	0	8	0	8
t786	6	0	0	6
SCC*mec*				
IVa	30	0	1	31
IVc	3	13	0	16
V	0	2	0	2
ND^*a*^	0	3	0	3
Negative	26	2	2	30

a ND, not determined.

In total, 64% (52/82) of the ST88 genomes harbored the *mecA* gene (MRSA), including 33 from humans, 18 from foods, and one from an animal sample. Most MRSA strains were from Africa (24/52) and Asia (20/52), with five from Europe and three from America. The predominant SCC*mec* type was SCC*mec*IVa (31/52), followed by SCC*mec*IVc (16/52) and SCC*mec*V (2/52). Nearly all human-associated MRSA strains were SCC*mec*IVa (30/33), with three isolates identified as SCC*mec*IVc. Meanwhile, most food-associated MRSA strains carried the SCC*mec*IVc element (13/18); two strains carried the type V SCC*mec* element, while three SCC*mec* element variants from food-associated MRSA strains were found to carry the class C2 *mec* gene complex (IS*431*-*mecA*-Δ*mecR1*-IS*431*) but to lack the *ccr* gene complexes (Fig. S2). The sole animal-associated MRSA strain was identified as SCC*mec*IVa (Fig. 1 and Data Set S1).

### Virulence factor-encoding genes in ST88 genomes.

All ST88 genomes contained 16 genes of the capsular serotype 8 (*cap8*) cluster (*cap8A* to *cap8P*); the immunity modulation genes *adsA* and *sbi*; the adhesin genes *ebp*, *map*, and *sdrC* (80/82) plus *sdrD* (74/82) and *sdrE* (76/82); the sortase B gene *srtB*; and the polysaccharide intercellular adhesion locus (*ica*) genes *icaA* to *icaD* and *icaR* (Fig. S3 and Data Set S1). Several isolates carried the adhesion genes *clfA* (30/82) and *clfB* (78/82), most of which were from humans (28/30 and 55/78, respectively). Genes encoding secreted enzymes were identified in all isolates, including those for aureolysin (*aur*), serine proteases (*sspA*, *sspB*, and *sspC*), lipase (*geh* and *lip*), and hyaluronidase (*hysA*).

Most ST88 genomes harbored hemolysin genes, including *hlb*, *hld*, *hlgA*, *hlgB*, *hlgC*, and *hly/hla* (except for 1 genome that was negative for *hly/hla*), which have been shown to play important roles in skin colonization and infection (26). The ST88 genomes also encoded a key virulence factor, type 7 secretion system (T7SS), which has been found to contribute to membrane integrity and homeostasis in the presence of antimicrobial fatty acids (27). All ST88 genomes also contained the seven iron uptake protein genes *isdA* to *isdG*, but most isolates lacked enterotoxin genes. Similarly, the gene encoding exfoliatin (*eta*) was found in only seven isolates, all from humans. Twenty-five strains (13 methicillin-susceptible S. aureus [MSSA] and 12 MRSA) were positive for the PVL genes *lukS* and *lukF*. In detail, 88% (22/25) were human-associated isolates (Thailand, *n* = 9; Tanzania, *n* = 7; Denmark, *n* = 4; China, *n* = 1; Lebanon, *n* = 1) and three were from food samples, all from China.

### Biofilm production.

**FIG 2:**
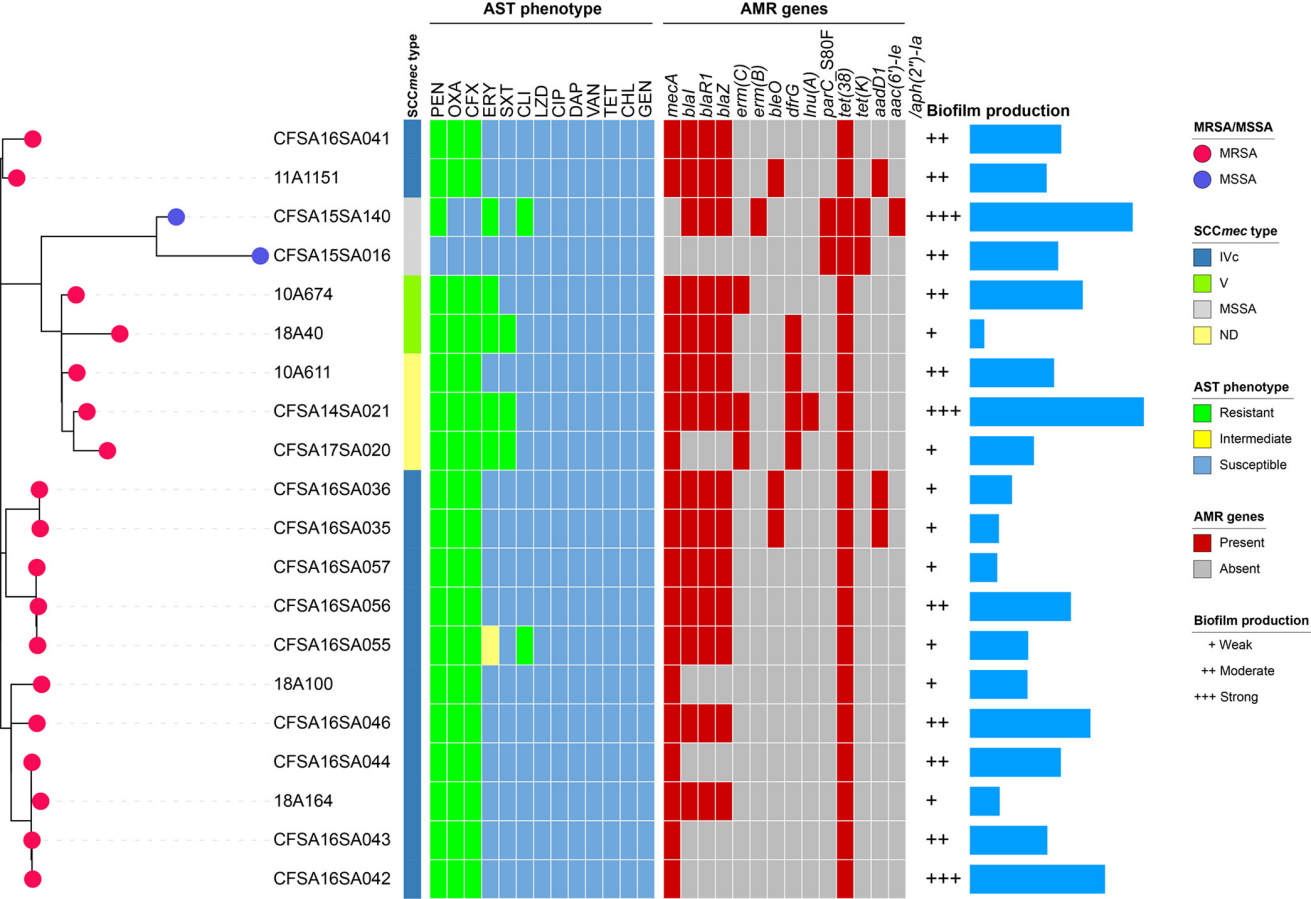
Antimicrobial-susceptible phenotype, presence/absence profile of antimicrobial resistance genes, and biofilm production of 20 Chinese ST88 S. aureus isolates collected in this study. Methicillin-resistant and -susceptible S. aureus isolates are indicated as red-pink (MRSA) or warm blue (MSSA) leaf nodes, respectively. The SCC*mec* types are indicated as colored columns. Resistant, intermediate, and susceptible phenotypes determined by antimicrobial susceptibility testing (AST) are indicated in green, yellow, and blue, respectively, as shown in the heatmap. Presence and absence of antimicrobial resistance genes are indicated in red and gray, respectively, as shown in the heatmap. The biofilm production of 20 isolates is shown with symbols (+ to +++) and a simple bar chart representing their capacity for biofilm formation. ND, not determined.

We evaluated biofilm production ability in the 20 isolates from our collection using M9 minimal medium at 37°C (Fig. 2). The results showed that, in addition to the negative control, all cultures in each microtiter plate well became suspended after incubation, indicating that all isolates could utilize glucose to grow in M9 medium. All of these 20 isolates could produce biofilms to various degrees of formation. Eight (40%, 8/20) could produce weak biofilms; nine (45%, 9/20) showed moderate biofilm formation; three (15%, 3/20) isolates had strong biofilm production ability. In addition, 8 of the 20 isolates (40%, 8/20) contained mutations within their biofilm-related genes (Data Set S1), including 4 with two point mutations at *icaB* (S154N) and *lip* (V161M), 2 with one point mutation at *hlgA* (Q168P), 1 with two point mutations at *icaD* (S89N) and *lip* (G309D), and 1 with one point mutation at *icaR* (I93N). However, we observed no significant correlations between these mutations and the biofilm-producing phenotype.

### Presence of prophages and virulence genes mediated by prophages.

We identified prophages in all 82 ST88 genomes by uploading them to the PHAge Search Tool Enhanced Release (PHASTER) server. In total, 48 types of prophages were detected; the top 3 were Staphy_StauST398_3 (87.8%, 72/82), Staphy_PT1028 (68.3%, 56/82), and Staphy_P282 (64.6%, 53/82). Of note, we found that all 20 food-associated ST88 genomes carried all three dominant prophages (Data Set S1).

**FIG 3:**
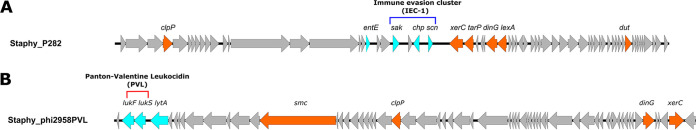
Genetic structure of prophages carrying virulence genes identified from ST88 isolates in this study. (A) Prophage Staphy_P282 (42.5 kb) carrying the immune evasion cluster IEC-1 genes including those encoding staphylococcal complement inhibitor (*scn*), chemotaxis-inhibiting protein (*chp*), and staphylokinase (*sak*) and the enterotoxin gene *entE*. (B) Prophage Staphy_phi2958PVL (49.4 kb) carrying the PVL genes (*lukF* and *lukS*). Genes are shown with the direction of transcription and color coded. The functional genes are shown as dull orange arrows, virulence genes as light blue arrows, and the hypothetical proteins as silver arrows.

We further evaluated the potential of prophages carrying virulence genes (Fig. 3). We found that the prophage Staphy_P282 (42.5 kb) carried the immune evasion cluster 1 (IEC-1) genes including those encoding staphylococcal complement inhibitor (*scn*), chemotaxis-inhibiting protein (*chp*), staphylokinase (*sak*), and enterotoxin (*entE*) (Fig. 3A). Other genes, such as *clpP* (encoding the proteolytic subunit of ATP-dependent Clp protease), *xerC* (tyrosine recombinase), *tarP* (β-*N*-acetylglucosaminyltransferase), *dinG* (3′-to-5′ exonuclease), *lexA* (LexA repressor), and *dut* (deoxyuridine triphosphatase), were also found in this prophage. In addition, the prophage Staphy_phi2958PVL (49.4 kb) harbored most of the *lukS* and *lukF* (PVL) genes (80%, 20/25), including 19 human- and 1 food-associated isolate (Fig. 3B). Downstream of this PVL gene cluster, the *lytA* gene encoding *N*-acetylmuramoyl-l-alanine amidase was identified in the same direction. The *smc* gene encoding the major tail protein of the TP901-1 family phage, as well as the *clpP*, *dinG*, and *xerC* genes, was also found in this prophage.

### AMR genes in ST88 genomes.

To understand how antimicrobial selection pressure drives ST88 evolution, we studied the existence and colocalization of antimicrobial resistance (AMR) genes in ST88 genomes (Fig. S3 and Data Set S1). In total, 50% (41/82) of the ST88 isolates were MDR (harboring AMR genes conferring resistance to ≥3 classes of antimicrobials), mainly encoding resistance to aminoglycosides, β-lactams, bleomycin, erythromycins, lincosamide, tetracyclines, and trimethoprim. β-Lactam resistance was mainly mediated by *mecA* (52/82, 61.0%), and 80.5% of isolates (66/82) harbored the penicillin-hydrolyzing class A β-lactamase gene (*blaZ*). We also identified penicillin-sensory transducing membrane protein (*blaR1*) and its corresponding repressor protein (*blaI*) from the genomes (both 81.7%, 67/82) (Fig. S3 and Data Set S1).

Other common AMR genes included *tet(38)* (100%) and *tet(K)* (29.3%, 24/82), encoding resistance to tetracycline; *dfrC* (24.4%, 20/82), encoding resistance to trimethoprim; *erm(C)* (15.9%, 13/82), encoding resistance to macrolides; and *vga(A)* (15.9%, 13/82), encoding resistance to lincosamide. We analyzed point mutations associated with quinolone resistance in S. aureus using AMRFinderPlus v3.9.8 (National Center for Biotechnology Information [NCBI], Bethesda, MD, USA). Two food-associated ST88 MSSA strains had the point mutation *parC* (S80F) in their quinolone resistance-determining regions (QRDRs). Furthermore, the ST88 isolates were also found to harbor resistant plasmids, with the genes *blaI*, *blaRI*, *blaZ*, *tet(K)*, and *erm(C)* frequently detected (Table S1).

The AMR profile of our 20 ST88 isolates is presented in Fig. 2 and Table S2. The most common resistance was found to penicillin (95.0%, 19/20), while all 18 MRSA strains were resistant to oxacillin and cefoxitin (90.0%, 18/20). Other phenotypes of resistance included those to erythromycin (25.0%, 5/20), trimethoprim-sulfamethoxazole (15.0%, 3/20), and clindamycin (10.0%, 2/20).

### Insertions of antimicrobial resistance determinants in SCC*mec* elements.

**FIG 4:**
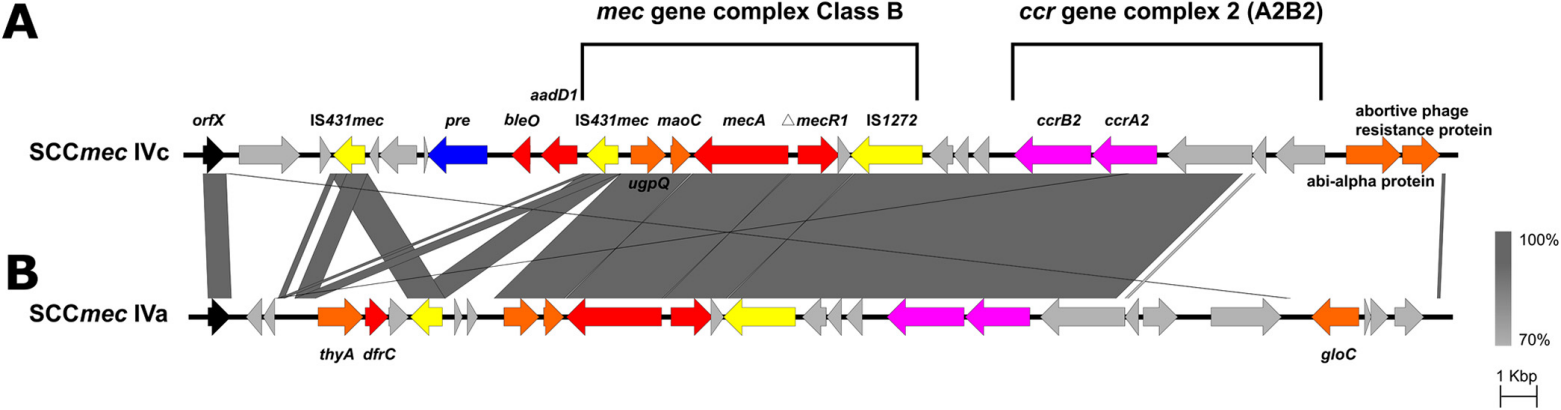
Overview of the SCC*mec* cassette with insertions of antimicrobial resistance genes in this study. (A) The SCC*mec*IVc cassettes identified from two Tanzanian genomes (GCA_900097575.1 and GCA_900097635.1). (B) The SCC*mec*IVa cassettes identified from four Chinese genomes (11A1151, CFSA16A035, CFSA16SA036, and GCA_001986135.1). Gray shading represents regions of homology. Genes are shown with the direction of transcription and color coded. The *orfX* gene is shown as black arrows, functional genes as dull orange arrows, plasmid-related genes as vibrant blue arrows, resistance genes as red arrows, *ccr* genes as pink arrows, insertion sequences as yellow arrows, and the hypothetical proteins as silver arrows.

Genome mining revealed that the SCC*mec* cassette could have extra AMR genes (Fig. 4). Resistance clusters containing *bleO* and *aadD1* with the plasmid recombination enzyme (*pre*) and IS*431mec* in the same direction were identified in the four SCC*mec*IVc elements upstream of the class B *mec* gene complex. In addition, we found that two SCC*mec*IVa elements in Tanzanian MRSA isolates harbored the *dfrC* gene, with other *dfrC* genes located elsewhere else on the chromosomes or plasmids.

### Phylogeographical context and comparative genomics of ST88 MRSA.

**FIG 5:**
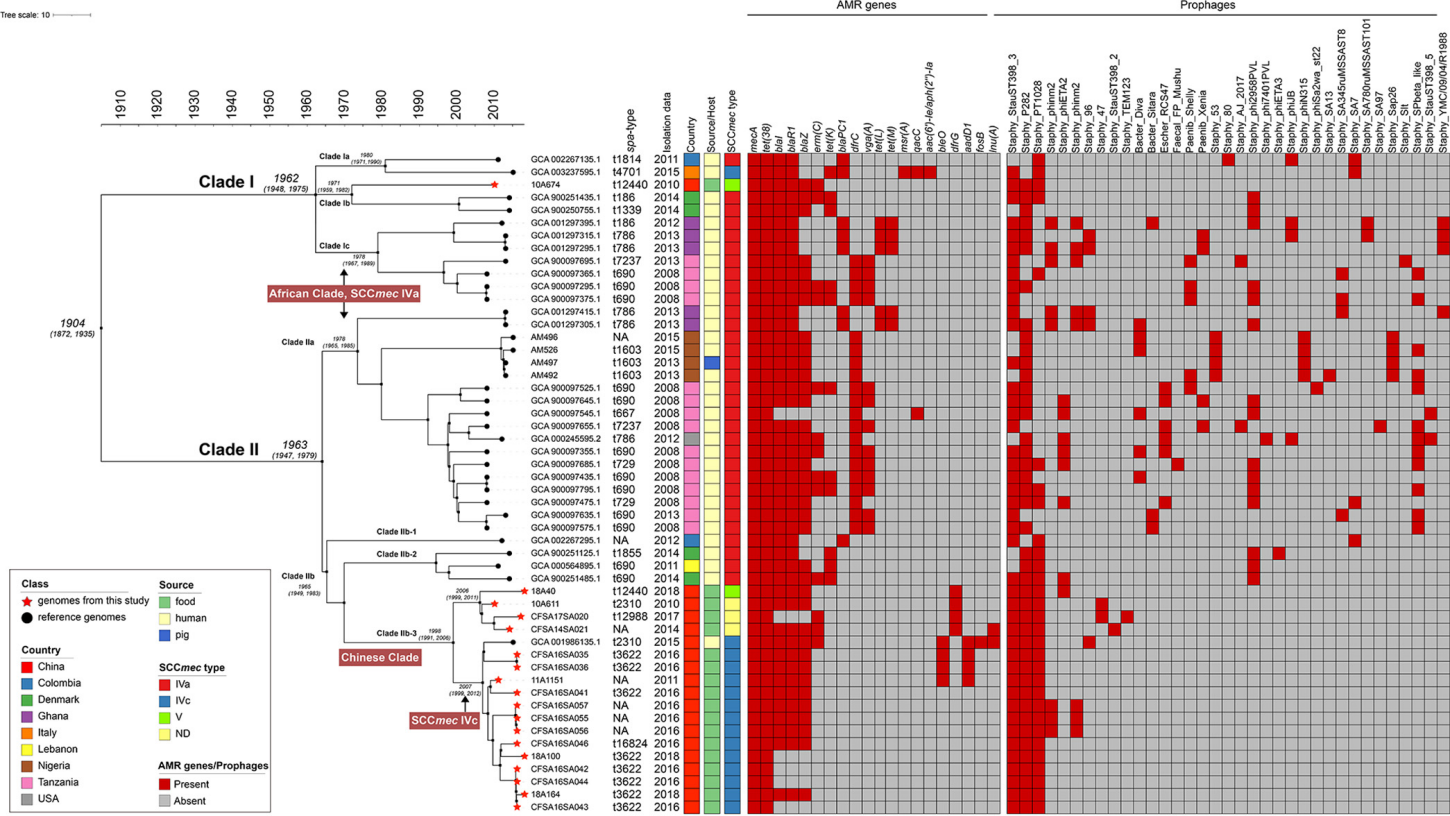
Bayesian phylogenetic analysis of 52 MRSA genomes. Isolates from our study and reference isolates are indicated as red star and black circle nodes, respectively. Colors in columns illustrate countries, sources and hosts, and SCC*mec* types. Presence and absence of antimicrobial resistance genes and prophages are indicated in red and gray, respectively, as shown in the heatmap. The African and Chinese clades, as well as relevant evolutionary events, are displayed in red boxes, and selected divergence time and 95% confidence intervals (CIs) are shown at the nodes.

We used Bayesian phylogenetic inference to decipher the evolutionary history of ST88 MRSA and to identify key genetic changes associated with its adaptation to different populations (Fig. 5). Pan-genome analysis identified 2,300 core genes across all 52 ST88 MRSA genomes, while the 30 MSSA genomes were excluded from the data set. The ST88 MRSA phylogeny exhibited a strong temporal signal (*R*^2^ = 0.5565), while the mean substitution rate was 2.83 × 10^−6^ (95% highest posterior density [HPD] interval, 2.65 × 10^−6^ to 3.01 × 10^−6^) substitutions per site per year. The most recent common ancestor (MRCA) of ST88 MRSA was estimated to be around 1904 (95% HPD, 1832 to 1965). Our analysis divided these 52 genomes into two major clades (I and II) (Fig. 5). The ancestral clade I (*n* = 12) originated around 1962 (95% HPD, 1928 to 2001), mixed with isolates from human (*n* = 11) and food (*n* = 1) sources. Meanwhile, the ancestral clade II (*n* = 40) originated around 1963 (95% HPD, 1933 to 2003), and its genomes were divided into two subclades (IIa and IIb). Clade IIa (*n* = 18) showed a pattern of divergence starting around 1978 (95% HPD, 1947 to 2004), while clade IIb (*n* = 22) showed a pattern of divergence starting around 1965 (95% HPD, 1941 to 2004).

Notably, the evolution of ST88 genomes possessed geographically specific characteristics (Fig. 5). All African MRSA strains (*n* = 24) were clustered into clades Ic and IIa, which were collectively labeled the African clade (mixed with 1 genome from the United States); all were subtyped as SCC*mec*IVa, with t690 (*n* = 10) the predominant *spa* type. Several AMR genes were solely detected in this clade, such as *dfrC* (*n* = 20, including 1 from the United States), *vga(A)* (*n* = 13), and *tet(L)* and *tet(M)* (both *n* = 5). In addition, all Chinese MRSA strains were clustered as the Chinese clade into clade IIb-3, with one exception that was mixed with two Danish MRSA strains in clade Ib. We estimated the Chinese ST88 MRSA strains to be present around the 2000s; divergence occurred when one clade acquired the SCC*mec*IVc element during ~2006 and ~2007. We found *spa* type t3622 (8/18) to dominate among the Chinese MRSA strains, and the AMR genes *aadD1* (*n* = 4), *bleO* (*n* = 4), *dfrG* (*n* = 4), *lnu(A)* (*n* = 2), and *fosB* (*n* = 1) were detected only in this clade. Furthermore, several prophages were also found only in the Chinese clade, such as Staphy_47 (*n* = 2), Staphy_StauST398_2 (*n* = 1), and Staphy_TEM123 (*n* = 1).

## DISCUSSIONS

The emergence of CA-MRSA with different genetic backgrounds has created a huge challenge to empirical therapy. The ST88 isolates have become the most common sequence type in several African countries, accounting for >50% of the MRSA isolates from clinical specimens (28). In China, although ST59 is still the dominant CA-MRSA clone, ST88 has frequently been detected in humans or food-producing animals (25). Accordingly, ST88 accounts for 8.8% to 16.3% of pediatric CA-MRSA infections in China (29, 30). It is also reported sporadically in the community and hospital settings in European and Latin American countries (31, 32). A comprehensive understanding of the epidemiology and spread of specific clones can help control MRSA infections. However, reports on the genomic characteristics of ST88 strains and their evolutionary histories are still insufficient. To this end, we described here the genomic context, AMR, biofilm production, and Bayesian divergence of ST88 isolates collected in China during 2010 to 2018, combined with the globally available ST88 genomes.

ST88 isolates have been previously detected from various sources (33, 34), but most ST88 genomes available to date are human relevant, and therefore a big knowledge gap exists for food-associated strains. Thanks to a large-scale investigation of foodborne S. aureus in China, we were able to collect 20 food-associated ST88 isolates. Unlike those of other countries, the Chinese ST88 isolates formed into two distinct clades; meanwhile, the *spa* types we identified were found solely from China, suggesting a restricted epidemic of ST88 clones in China. Furthermore, the Chinese isolates of human- and food-associated ST88 were clustered on the same branch of the phylogenetic tree, and isolates from other countries showed similar patterns. These observations indicated that the ST88 strains might occur in exchanges between humans and foods or animals, posing a nonnegligible threat to food safety and to human and animal health, as has been demonstrated in previous research (33, 34).

Our data highlighted the diverse genetic backgrounds of the ST88 isolates as shown by *spa* typing. The main *spa* types, t690 and t786, in the human ST88 isolates in this study have been reported as dominant clones that cause human infections (35, 36). In addition, ST88-t786 and other ST88 clones of t186, t1603, and t729 have frequently been detected in humans, food. or food animals (33, 37–39). This further suggests potential transmission among the above-mentioned hosts. In addition, the major identified food-associated clone ST88-t3622 has been also reported in clinical cases of infection (40, 41). Therefore, these food-associated ST88 clones constitute a major concern in China that could quickly spread and lead to widespread infections among humans.

MRSA is considered a major cause of hospital- and community-acquired infections. Most ST88 MRSA genomes in this study were from Africa (24/52) and China (19/52), with SCC*mec*IVa and SCC*mec*IVc the predominant SCC*mec* elements, respectively. Meanwhile, we identified two isolates as type V SCC*mec* elements. Both SCC*mec*IV and -V are small, which has been attributed to fewer metabolic burdens of protein synthesis during replication (42). This feature might be a prerequisite for ST88-MRSA to successfully colonize humans and animals in the environment. Moreover, a rising number of SCC*mec* variants that harbor composite cassettes and pseudo-SCC*mec* elements but lack *ccr* genes have been detected in recent years (43, 44). In this study, we also identified three SCC*mec* variants from our Chinese collection that carried only the *mec* gene complex (class C2) while lacking the *ccr* gene complexes, suggesting the ongoing evolution of ST88 MRSA.

Virulence is an important determinant of whether bacteria will induce clinical infections (45). Regarding the risk of pathogenicity, we assessed the presence of virulence factors among all 82 ST88 genomes in this study. Notably, most ST88 isolates harbored virulence genes related to immune modulation, adherence, exoenzyme, effector delivery system, and hemolysin, all of which are core virulence genes. These virulence genes might therefore play roles in host adaption and evolution of virulence in the ST88 lineage. Notably, only a few ST88 isolates (6/82) harbored the classic staphylococcal enterotoxin (SEA to SEE) determinants that cause sporadic food-poisoning incidents or even FBOs (46). This finding, which was inconsistent with previous reports (24, 25), suggested that the most threatening of ST88 isolates in humans cause infections rather than food poisoning. Moreover, previous research has revealed that ST88 can produce very strong biofilms (18). We report that the adhesion-associated genes *ebp* and *ica* and exotoxin genes *hly/hla*, *hlg*, *lip*, and *lukD*, which have been verified to contribute to biofilm formation (18, 24), were found in almost all 82 genomes in this study. Therefore, the ability to form biofilms could help S. aureus to persist in infections in both subclinical and clinical cases (47, 48). In this study, all 20 Chinese ST88 isolates could form biofilms as determined by microtiter plate assay. The high incidence of biofilm-producing S. aureus isolates in this study suggests the need for the food industry to improve its quality assurance systems in order to decrease and eliminate these isolates. Although we identified point mutations in biofilm-related genes in this study, we observed no significant correlations between these mutations and the biofilm-producing phenotype. Further studies are needed to reveal the differences in the biofilm production ability.

Prophages, as mobile genetic elements (MGEs), are repressed and integrated into the host chromosomes that encode virulence factors such as superantigens, enterotoxins, PVLs, and biofilm formation (45, 49, 50). These MGEs have a high clinical relevance (51). In this study, we found that two prophages, Staphy_P282 and Staphy_phi2958PVL, possessed virulence genes such as the IEC-1 and PVL clusters, respectively. The IEC-1 cluster included three genes implicated in immune evasion, *scn*, *chp*, and *sak*, which play major roles in complement evasion: (i) *scn* binds to C3 convertases, preventing the activation of all three complement pathways (52); (ii) *chp* binds to complement C5a receptor 1 (C5aR1) and formyl peptide receptor 1 (FPR1), blocking recognition of C5a and *N*-formylmethionyl-leucyl-phenylalanine (fMLF) chemoattractant (53); and (iii) *sak* activates plasminogen into plasmin, a serine protease bound to the staphylococcal membrane which disrupts opsonization and phagocytosis and blocks the cytolytic effect of human alpha-defensins (54). Therefore, the IEC-1 cluster encoding prophage Staphy_P282 might play a role in ST88’s specific mechanisms of human adaptation. PVL is a bicomponent, synergohymenotropic toxin that exerts cytolytic pore-forming activity directed at the cell membranes of neutrophils, monocytes, and macrophages (55). Clinically, it is associated with skin abscesses and necrotizing pneumonitis (56, 57). PVL genes are usually found in only 2% of S. aureus clinical isolates but have been found in most CA-MRSA strains (58, 59). In the current study, >30% (25/82) of the ST88 isolates were positive for PVL, which can worsen the infections in humans, and 80% (20/25) were found in the Staphy_phi2958PVL prophage. Phage dynamics might cause conversion between commensalism and pathogenicity (60, 61). This finding of food- and human-associated PVL-encoding prophages indicated the potential dissemination of ST88 between humans and the food chain.

In recent years, the emergence of MDR S. aureus, particularly MRSA, leading to animal and human infections, as well as transmission and persistence in the environment, has become a growing public-health concern (62–64). In the current study, all ST88 isolates carried AMR genes, while 50% (41/82) harbored MDR genes. More than 80% of ST88 isolates carried the penicillin resistance-encoding genes *blaZ*, *blaR1*, and *blaI* (65). We subsequently found these three AMR genes to be plasmid carried, which could potentially be the origin of chromosome-borne resistance genes through recombination. Moreover, we also frequently detected the tetracycline efflux pump-encoding genes *tet(38)* and *tet(K)*, the trimethoprim-resistant dihydrofolate reductase gene *dfrC*, the 23S rRNA (adenine(2058)-N(6))-methyltransferase *erm(C)*, and the ATP-binding cassette transporter F (ABC-F) type ribosomal-protection protein Vga(A)-encoding gene *vga(A)* in the ST88 genomes using AMRFinderPlus (66). The AMR test of our 20 ST88 isolates showed that 20% (4/20) were MDR, which was similar to the finding of previous reports from clinics or food in China (24, 67). However, the MDR rate in this study was much lower than that shown by our previous data from retail food (most were raw meat; MDR rate = 57.5%) (68).

As discussed above, SCC*mec* variants have been frequently detected in recent years (43, 44). Resistance can also occur by gene acquisition in the SCC*mec* elements (69, 70). Given the high importance of AMR in S. aureus, we tested for the presence of specific AMR genes in these SCC*mec* elements and found six genomes to have extra AMR genes in addition to the *mecA* gene, including 4 Chinese SCC*mec*IVc isolates (3 food associated and 1 human associated) enriched with genes for bleomycin (*bleO*) and aminoglycoside (*aadD1*) resistance (71, 72) and 2 Tanzanian SCC*mec*IVa isolates that showed insertion of the trimethoprim resistance gene (*dfrC*) (73). Our finding suggested that the high usage of antimicrobials in livestock and humans, as well as the potential dissemination risk of AMR among humans, animals, foods, and the environment, might promote the variability and complexity of AMR.

The combination of phylogenetic and molecular clock analysis provided a compelling depiction of the ST88-MRSA’s emergence from its proposed origins around 1904 to its current status as the major CA-MRSA lineage in Africa and Asia, especially China (15, 16). Bayesian divergence analysis highlighted that the African ST88-MRSA strains emerged in the background by acquiring the SCC*mec*IVa element. These strains were clustered into two clades, clades Ib and IIa, indicating the individual evolutionary paths along which both clades have developed. Although the CA-MRSA strains have been reportedly increasing since the 1990s, the African ST88-MRSA strains were estimated to have emerged around 1978, and this is consistent with the earliest report (also around 1978) of MRSA in Africa (74). Recent research has demonstrated that the outbreak strain ST88-MRSA-IVa in Europe was likely imported from Africa (35). In this study, the European ST88-MRSA-IVa clone shared a similar divergence date with the African ones (clade I), suggesting that this African clone might have been transmitted to Europe much earlier. Moreover, one U.S. isolate was found to be clustered within the African clade IIa, suggesting another potential transmission of this clone from Africa to the United States. Furthermore, the ST88-MRSA strains from China (clade IIb-3) in our cohort tended to form independent clusters with other countries, implying geographical differentiation. The Chinese ST88-MRSA isolates showed more-recent diversification (around the 2000s), which might have been driven by economic growth and increased antimicrobial use (AMU) (75, 76). The estimated divergence date of the Chinese ST88-MRSA is consistent with the first report of CA-MRSA in China in 2002, and with the significantly increased carriage rate of CA-MRSA from <1% in 2001 to 2005 to 4% in ~2008 (77, 78). In addition, Bayesian divergence analysis suggested that the acquisition of different SCC*mec* elements (SCC*mec*IVc, SCC*mec*V, and nontypeable SCC*mec* elements) could explain the main divergence in the Chinese ST88-MRSA in ~2006 and ~2007. Finally, specific characteristics were identifiable in the ST88-MRSA genomes from Africa and China, such as the enrichment of different SCC*mec* elements, *spa* types, AMR genes, and prophages, indicating different host adaption mechanisms of ST88-MRSA in these two regions. Similar observations have been described from other MRSA clones (79, 80), although the driving factor remains unclear and deserves future studies.

In summary, our results revealed the evolution of ST88, a very common community-associated clone that spreads among humans, animals, and foods in Africa and Asia and sporadically spreads in other parts of the world. Our food-associated ST88 genomes will remedy the lack of food-associated ST88 genome data in current research. The characteristics of possession of specific *spa* types and clustering in two distinct clades on the phylogenetic tree suggested a restricted epidemic of ST88 clones in China. The phylogenetic tree also indicated potential exchanges between humans and foods or food animals. The ST88 isolates were rich in virulence genes related to infections but not to food poisoning and exhibited a high incidence of biofilm production, revealing their typical pathogenicity in hosts. The presence of IEC-1- and PVL-encoding prophages could play a role in ST88 as specific mechanisms of human adaption and poses a serious public health risk. MDR genes, especially of plasmid origins, and SCC*mec* variants lacking *ccr* gene complexes or carrying insertions of AMR determinants can provide further avenues of investigation into the evolution of ST88 isolates. Bayesian phylogenetic analysis revealed geographical differentiation in the evolution of ST88 MRSA between Africa and Asia, with a more recent clonal evolution of ST88 MRSA in China. In accordance with the One Health concept, our study emphasized the importance of a large-scale working approach toward humans, foods, food animals, and related sectors in order to investigate the epidemiological transmission and evolutionary dynamics, as well as the virulence and resistance mechanisms, of ST88 in Africa, Asia, and elsewhere. Effective surveillance and monitoring of AMU, as well as implementation of good hygiene management in the human and animal communities and in the food industry, are recommended measures to control the emergence and spread of this bacterium.

## MATERIALS AND METHODS

### Bacterial isolation.

Twenty ST88 S. aureus isolates, collected in eight Chinese provinces from 2010 to 2018, were included in this study. Another set of 62 ST88 S. aureus sequences was retrieved from the NCBI GenBank comprising publicly available genomes at the time of this study (June 2021). To retrieve the ST88 genomes, we downloaded approximately 18,000 S. aureus genomes from the NCBI GenBank, and the multilocus sequence type (MLST) tool (https://github.com/tseemann/mlst) was used to identify the sequence types of each genome.

### WGS and analysis.

Whole-genome sequencing (WGS) of 20 ST88 isolates was performed by Beijing Novogene Bioinformatics Technology Co., Ltd. (Beijing, China), using an Illumina NovaSeq PE150 sequencer (Illumina, Inc., San Diego, CA, USA). Clean data were filtered using Trimmomatic (https://github.com/usadellab/Trimmomatic) (81) and assembled using SPAdes v3.14 (http://cab.spbu.ru/software/spades/) (82). Genomes were annotated with Prokka v1.14.5 (https://github.com/tseemann/prokka) using the default parameters with -addgenes -usegenus (83). We identified and extracted plasmid-derived contigs from the assemblies using the MOB-Suite v2.0.0 (https://github.com/phac-nml/mob-suite) (84). AMR genes were identified using AMRFinderPlus v3.9.8 (66). We identified virulence genes using ABRicate v1.01 (https://github.com/tseemann/abricate) and the Virulence Factor Database (VFDB; http://www.mgc.ac.cn/VFs/main.htm) with 90% identity and 75% query coverage as cutoffs. SCC*mec* and *spa* type analyses were conducted using SCCmecFinder v1.2 (85) and spaTyper 1.0 (86), respectively. The PHAge Search Tool Enhanced Release server (PHASTER; http://phaster.ca/) was used to detect prophages in the genomes (87, 88). We compared nontypeable SCC*mec* elements, SCC*mec* elements possessing extra resistance genes, and identified prophages from the assembled chromosomes, respectively, and visualized them using Easyfig v2.2.2 (89).

### Core gene alignment and phylogenetic analysis.

All annotated files were used for pan-genome analysis with core gene alignments via Roary v3.11.2 (http://sanger-pathogens.github.io/Roary/) (90). We used the snippy tool (https://github.com/tseemann/snippy) to remove the recombinant regions from the core genome alignment. FastTree v2.1 (https://anaconda.org/bioconda/fasttree) was used to construct the phylogenetic trees, using the generalized time-reversible (GTR) replacement model with CAT approximation (91). We subsequently visualized the trees using Interactive Tree of Life (iTOL) v4 (https://itol.embl.de/) (92).

### Bayesian divergence estimates.

ST88 MRSA’s divergence data were estimated using Bayesian Evolutionary Analysis by Sampling Trees (BEAST) v1.10.4 (https://beast.community) (93). We analyzed the core genome alignment of all 52 available ST88 MRSA strains using Roary v3.11.2 (90). The TempEst v1.5.3 was used to check the temporal signal. To investigate the temporal signal in the data set, we analyzed the correlation between root-to-tip genetic distance and year of sampling on the maximum-likelihood tree using TempEst (94). The best-fitting model priors were defined by testing the combination of four clock models (strict, uncorrelated relaxed, random local, and fixed local), three tree priors (constant size, Bayesian skyline, and birth-death process), and two substitution models (Hasegawa-Kiahino-Yano [HKY] and generalized time-reversible [GTR]). The best model was a fixed local clock model, with a constant size model and a GTR-gamma nucleotide substitution model. Analysis was performed with two independent chains until the effective sample size (ESS) for all parameters exceeded 200 per chain. This entailed each chain running for approximately 60 million steps. Convergence was assessed in Tracer v1.7.1 (95). We selected the maximum clade credibility tree using TreeAnnotator v1.10.4 (https://beast2.blogs.auckland.ac.nz/treeannotator/) (96) and then visualized it in iTOL v4 (92).

### Biofilm production.

Biofilm formation was assessed in a 96-well microtiter plate assay using minimal medium M9 (6 g/L Na_2_HPO_4_, 3 g/L KH_2_PO_4_, 0.5 g/L NaCl, 1 g/L NH_4_Cl, 2 mM MgSO_4_, 0.1% glucose, and 0.1 mM CaCl_2_) as described previously (97). After overnight growth in tryptone soy broth medium (TSB; Oxoid Ltd., Basingstoke, UK), 200 μL of cell suspension diluted to 1:100 (approximate optical density at 600 nm [~OD_600_] = 0.1) was transferred into each microtiter plate well, and the latter was incubated at 37°C for 72 h. After three brief washes with 200 μL phosphate-buffered saline (PBS) solution and a 20-min fixation step with 200 μL methanol, all plates were stained with 200 μL 0.4% (wt/vol) crystal violet (CV) for 15 min and washed with 200 μL PBS for another 15 min. The formed biofilm was then dissolved with 200 μL 33% (wt/vol) acetic acid for 30 min. The biofilm formation was measured at 570-nm optical density (OD) in a microtiter plate reader (Tecan, Mannedorf, Switzerland). Salmonella enterica serovar Typhimurium ATCC 14028, a strong biofilm-forming strain, was selected as the positive control, and sterile TSB was used as the negative control for the biofilm production assays. An OD_570_ value of 0.6 was applied as the cutoff to distinguish between biofilm producers and non-biofilm producers (cutoff [ODc] = average OD plus 3 standard deviations [SD] of negative control). The biofilm formation was classified as strong, +++ (OD_570_ > 1.8); moderate, ++ (1.8 > OD_570_ > 1.2); weak, + (1.2 > OD_570_ > 0.6); and negative, − (OD_570_ < 0.6).

### Antimicrobial susceptibility testing.

Antimicrobial susceptibility testing of the 20 ST88 S. aureus isolates was evaluated using the broth dilution method with the Biofosun Gram-positive panels (Shanghai Biofosun Biotech, Shanghai, China) per manufacturer’s instructions. MICs were interpreted using the Clinical and Laboratory Standards Institute (CLSI) guidelines (98). S. aureus strains ATCC 29213 and ATCC 25923 were used as quality controls.

### Data availability.

The GenBank accession numbers for all 82 isolates used in this study are listed in Data Set S1 in the supplemental material.
