# Supplementary material for: Molecular Evolution and Genomic Insights into Community-Acquired Methicillin-Resistant Staphylococcus aureus Sequence Type 88
Source: Microbiol Spectr. 2022 Jun 22;10(4):e00342-22. doi: 10.1128/spectrum.00342-22 (PMC9430171; doi:10.1128/spectrum.00342-22)
Supplement: Supplemental file 2 — Supplemental material. Download spectrum.00342-22-s0002.pdf, PDF file, 1.4 MB [file spectrum.00342-22-s0002.pdf]

Table S1 Antimicrobial resistance genes of plasmid origin among 82 ST88 genomes identified by Mob-Suite

| Antimicrobials | Resistance genes | No. of isolates |
|---|---|---|
| β-lactamase | *blaI* | 61 |
| | *blaR1* | 61 |
| | *blaZ* | 60 |
| | *mecA* | 10 |
| | *mecR1* | 7 |
| tetracycline | *tet(K)* | 24 |
| | *tet(38)* | 6 |
| | *tet(L)* | 5 |
| | *tet(M)* | 5 |
| macrolides | *erm(C)* | 13 |
| | *erm(A)* | 2 |
| | *erm(B)* | 1 |
| | *msr(A)* | 1 |
| lincosamide | *lnu(A)* | 4 |
| | *vga(A)* | 1 |
| bleomycin | *bleO* | 4 |
| chloramphenicol | *catA* | 4 |
| aminoglycoside | *ant(9)-Ia* | 2 |
| | *aac(6')-Ie/aph(2'')-Ia* | 2 |
| trimethoprim | *dfrC* | 2 |
| | *dfrG* | 2 |
| fosfomycin | *fosB* | 1 |

Table S2 Antimicrobial susceptibility testing of 20 ST88 isolates collected from China in this study, %(n)

| Antimicrobials | Resistant | Intermediate | Susceptible |
|---|---|---|---|
| penicillin | 95.0 (19) | 0.0 (0) | 5.0 (1) |
| oxacillin | 90.0 (18) | 0.0 (0) | 10.0 (2) |
| cefoxitin | 90.0 (18) | 0.0 (0) | 10.0 (2) |
| erythromycin | 25.0 (5) | 5.0 (1) | 70.0 (14) |
| trimethoprim-sulfamethoxazole | 15.0 (3) | 0.0 (0) | 85.0 (17) |
| clindamycin | 10.0 (2) | 0.0 (0) | 90.0 (18) |
| daptomycin | 0.0 (0) | 0.0 (0) | 100.0 (20) |
| linezolid | 0.0 (0) | 0.0 (0) | 100.0 (20) |
| ciprofloxacin | 0.0 (0) | 0.0 (0) | 100.0 (20) |
| vancomycin | 0.0 (0) | 0.0 (0) | 100.0 (20) |
| tetracycline | 0.0 (0) | 0.0 (0) | 100.0 (20) |
| chloramphenicol | 0.0 (0) | 0.0 (0) | 100.0 (20) |
| gentamicin | 0.0 (0) | 0.0 (0) | 100.0 (20) |

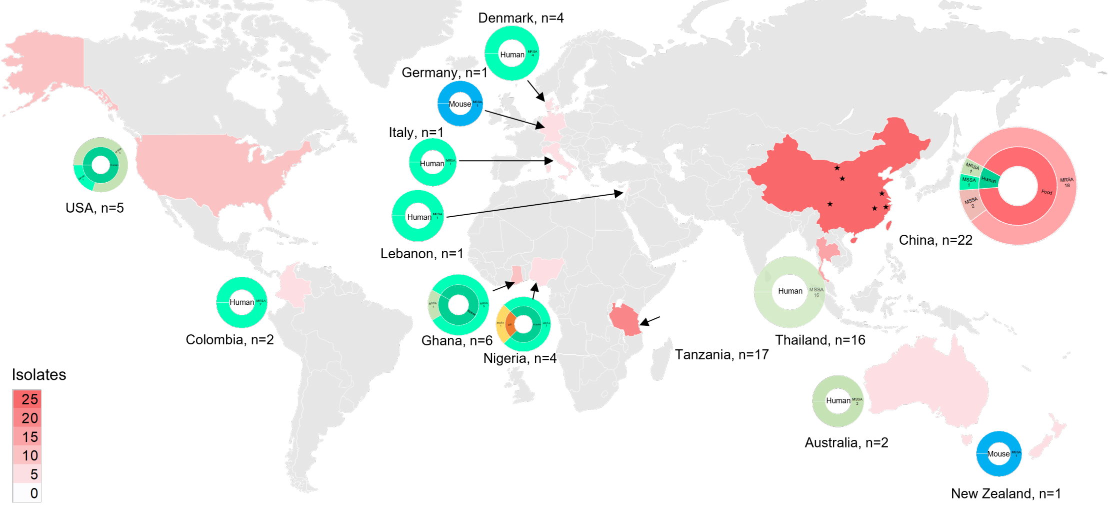


**Figure S1. World map showing number of the ST88 *S. aureus* isolates from China and other countries.** The countries with available genomes included in this study are colored from light pink to dark peach representing numbers of the *S. aureus* isolates. The sources or hosts, as well as numbers of MRSA and MSSA from each country are shown in pie charts.

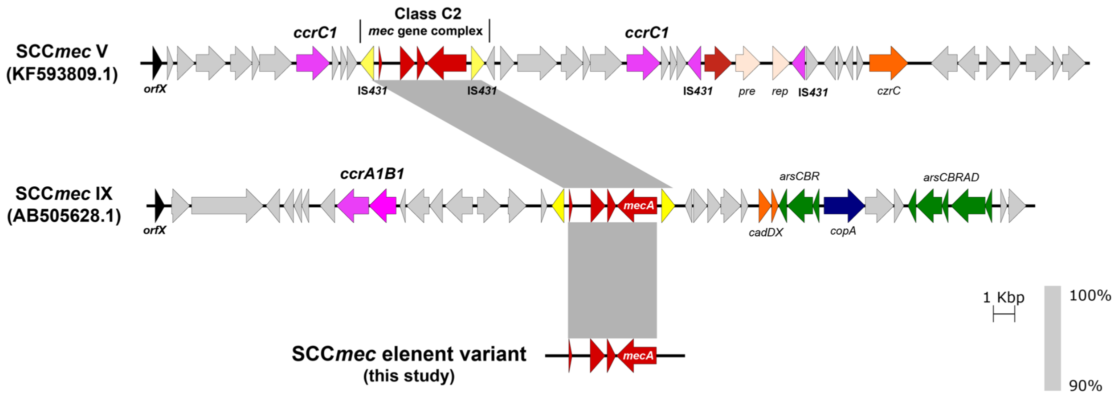


**Figure S2. Overview of SCC*mec* element variants from three food related MRSA (10A611, CFSA14SA021 and CFSA17SA020) collected in China, compared with the reference type V and XI SCC*mec* cassettes.** Grey shading represents regions of homology between the *mec* gene complexes from this study and reference SCC*mec* cassettes. Genes are shown with the direction of transcription and colour coded.

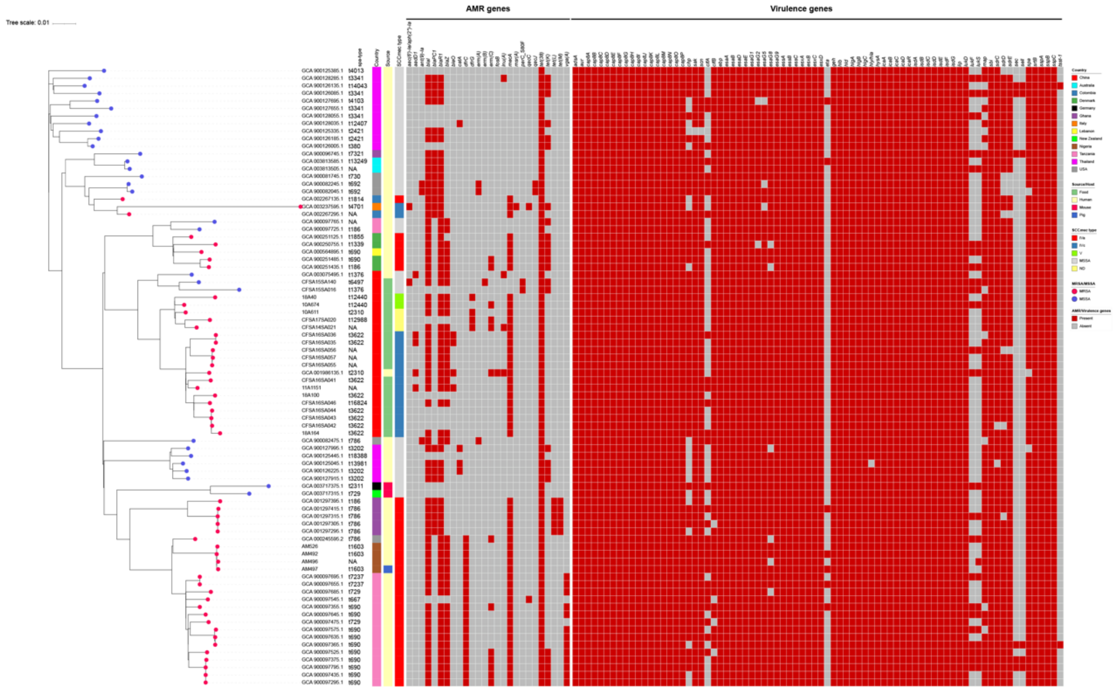


**Figure S3. Presence and absence of antimicrobial resistance and virulence genes identified from all 82 ST88 *S. aureus* isolates.** The MRSA and MSSA are indicated as red pink (MRSA) or warm blue (MSSA) leaf nodes, respectively. The countries, sources/hosts, and SCC*mec* types are shown in colour columns. Presence and absence of antimicrobial resistance genes and virulence genes are indicated in red and grey, respectively, as shown in the heatmap.
